# Limited utilization of serologic testing in patients undergoing duodenal biopsy for celiac disease

**DOI:** 10.1186/1471-230X-13-156

**Published:** 2013-11-09

**Authors:** Homer O Wiland, Walter H Henricks, Thomas M Daly

**Affiliations:** 1 Robert J. Tomsich Pathology and Laboratory Medicine Institute, Cleveland Clinic, Clinical Pathology, LL3-3, 9500 Euclid Avenue, 44195 Cleveland, OH, USA

**Keywords:** Celiac disease, Serology, Duodenal biopsy, Utilization

## Abstract

**Background:**

Clinical algorithms for the workup of celiac disease often recommend the use of serologic assays for initial screening, followed by duodenal biopsy for histologic confirmation. However, the majority of duodenal biopsies submitted to pathology for “rule out celiac” are negative. The objective of this study was to determine the underlying causes for this low diagnostic yield.

**Methods:**

We performed a retrospective review of pathology reports from 1432 consecutive duodenal biopsies submitted for pathologic assessment to “rule out celiac” and correlated biopsy results with results for concurrent serologic testing for celiac autoantibodies.

**Results:**

The majority of patients had no record of serologic testing prior to biopsy, and evidence of positive serology results was found in only 5% of patients. Most duodenal biopsies were submitted as part of a multi-site GI sampling strategy that included biopsies from other locations. In this context, serologic results correlated with the likelihood of significant duodenal and non-duodenal findings, and were also helpful in evaluating patients with indeterminate duodenal histology.

**Conclusions:**

The presence of a positive screening test for celiac autoantibodies does not appear to be a major driver in the decision to submit duodenal biopsies for evaluation of celiac disease, which accounts for the low incidence of findings in these samples. In patients where celiac serology testing was performed, the results were a good predictor of the likelihood of findings on biopsy.

## Background

Celiac disease is one of the most common autoimmune diseases, with an estimated prevalence of approximately 1% in various populations [1-3]. The disease is caused by an autoimmune response to gluten which leads to progressive villous atrophy in the small bowel, resulting in malabsorption. Gastrointestinal (GI) symptoms can be relatively nonspecific, such as diarrhea and abdominal pain. Systemic complications are common, and can include iron deficiency anemia and fatigue. Accurate recognition and diagnosis of celiac disease is important because implementation of a gluten-free diet can ameliorate many symptoms. If left untreated, celiac disease is associated with increased mortality in adult life from a range of causes, including autoimmune diseases and malignancy [4,5].

For patients with an appropriate clinical history, diagnostic tools for the workup of celiac disease can be divided into three categories; serologic assays to measure celiac-associated autoantibodies, genetic assays to identify HLA-DQ2 or -DQ8, and duodenal biopsy to document the presence of villous atrophy. Although many groups have published guidelines on the diagnosis and management of celiac disease and the role of testing in this process [6,7], surveys have found that there can be significant variation in adherence to these guidelines in different practice settings [8]. While the exact steps of the algorithms can vary slightly depending upon the specific population being tested, most approaches recommend using serologic assays either prior to duodenal biopsy [9,10] or concurrently with biopsy in cases with a strong clinical suspicion [11].

The most commonly-used serologic assays measure autoantibodies against tissue transglutaminase (tTG), deamidated gliadin (dGDN), and endomysial tissue (EMA). Antibodies against native gliadin are losing popularity because of inferior performance when compared to the newer dGDN assays [12,13]. Although most assays measure IgA antibodies against these targets, IgG versions are also available for use in patients with IgA deficiency, a disorder commonly associated with celiac disease [14]. The diagnostic characteristics of celiac serology tests have been well-described in many populations, and in general show analytical performance sufficient for use as a screening test [15-18]. tTG-IgA and EMA-IgA assays have shown the best diagnostic performance in most studies, with pooled sensitivities of 89- 90% and specificities of 98 – 99% in a recent systematic review of the literature [16]. Recent studies have suggested that the use of serologic testing prior to endoscopy could potentially reduce the need for intestinal biopsy to diagnose celiac disease [19].

Given the high sensitivity and specificity of serologic testing, one would expect to find a fairly high diagnostic yield in duodenal biopsies for celiac disease. In a population with a disease prevalence of 1%, a test with the characteristics described above (90% Sn, 98% Sp) would have an expected positive predictive value (PPV) of roughly 47%. However, the historical experience at our institution has been that the majority of duodenal biopsies submitted for “rule out celiac” are histologically normal. In an effort to understand the causes for this discrepancy, we retrospectively examined the utilization of celiac serology in a cohort of patients who had been sent for duodenal biopsy.

## Methods

### Case finding

**Figure 1:**
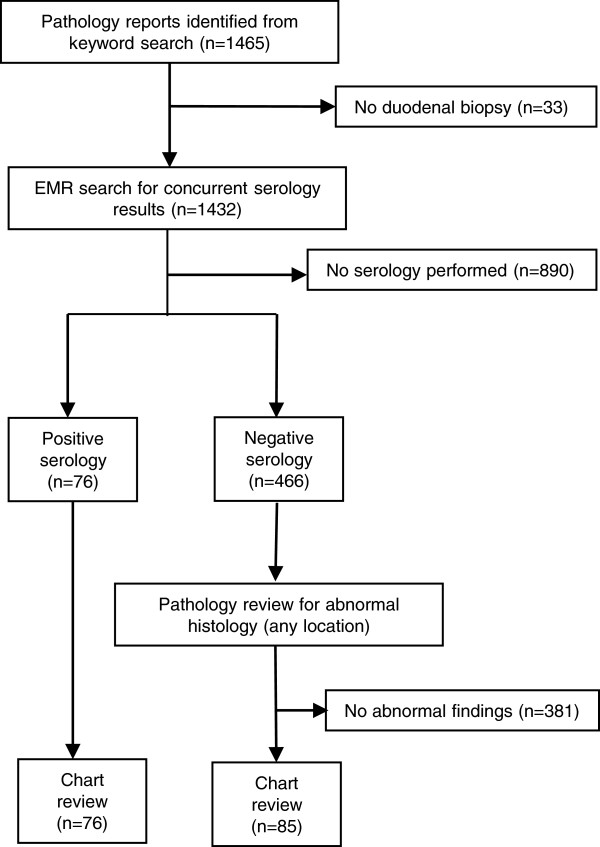
**Flow chart of data analysis.** Initial EMR searches for pathology reports and serology results were performed using the criteria described in methods. Serology totals include patients with either pre- or post-biopsy testing.

An automated query was run on the pathology laboratory information system (CoPath, Cerner Corp, Waltham MA) to identify any biopsy submissions that contained the words “celiac”, “gluten”, or “sprue” in the clinical data field (which is the field completed by the ordering physician to describe the reason for the submission). Case finding and subsequent chart review were performed following protocol approval by the institutional review board of the Cleveland Clinic Foundation. All biopsy specimens were initially reviewed and signed out by one of twelve pathology staff members belonging to the subspecialty gastrointestinal pathology group at the Cleveland Clinic, each of whom has fellowship training in gastrointestinal pathology or extensive experience in the field. Only samples with adequate material for a final report to be issued were included in the analysis. 1465 unique patients were identified during the 6 month period covered by the study (Figure 1). A manual review of reports eliminated 33 patients without duodenal biopsies, leaving 1432 for final review. Gender ratio of this cohort was 34:66 M:F, with a median age of 45 (IQR 24–61). Histologic findings as reported by the gastrointestinal pathologist who signed out the original specimen were classified into one of four categories; villous atrophy consistent with celiac disease (CD), intact villous architecture with increased intraepithelial lymphocytes (IVA-IEL) [20,21], normal duodenum, or other findings. Samples were not classified in a graded system (such as the Marsh score). Additionally, we examined reports to determine if biopsies were simultaneously submitted from sites other than the duodenum, and what additional findings were present in those sites.

### Serologic testing

An automated electronic medical record search was performed to identify any celiac serology results for the 1432 patients identified above. Serologic assays included in the search were tTG (IgA or IgG), dGDN (IgA or IgG) and EMA (IgA only). tTG IgA and IgG antibodies were measured using the respective QUANTA Lite h-tTG ELISA assays (INOVA Diagnostics, San Diego, CA), which utilize human RBC-derived tTG as the capture antigen. dGDN IgA and IgG antibodies were measured using QUANTA Lite Gliadin II ELISAs (INOVA Diagnostics, San Diego, CA), which utilize purified gliadin peptides for capture. The EMA IgA assay was performed using an indirect immunoflourescent assay (INOVA Diagnostics, San Diego, CA) with primate distal esophagus as the slide substrate. Total IgA results were also included in the routine panel to identify patients with IgA deficiency. All tests were performed in-house at the Cleveland Clinic as part of routine clinical testing in accordance with manufacturer’s recommendations. Patients with a non-negative result for any of the four assays were classified as having positive serology in the analyses that follow. tTG, dGDN, and EMA antibodies were detected in 51%, 54%, and 28% of patients with positive serologic results, respectively.

### Chart review

Clinical records were reviewed for the subset of 161 patients with either positive serology or biopsy results. In the cases reviewed the following information was noted: previous history of celiac disease, consumption of a gluten free diet, timing of the gluten free diet (before or after biopsy/serology), response to gluten free diet, HLA testing results, and the final clinical diagnosis. Patients were considered to have a diagnosis of celiac disease based on positive biopsy or serologic results in the context of appropriate clinical findings (such as response to gluten-free diet or prior history of CD). Finally, endoscopy reports were reviewed in the patients where serologic testing was performed to identify the indication for endoscopy.

## Results

During the 6 month period of the study, 1432 duodenal biopsies were received where celiac disease was part of the differential specified in the pathology request. Less than one-third of these patients had evidence of celiac serology results in the medical record, and even in patients with pre-biopsy testing the majority were serologically negative (Table 1). Celiac disease was noted as an indication for endoscopy in 11% of patients where serologic testing was performed (Table 2). Although only 5% (68/1432) of the study cohort had evidence of positive serologic results prior to biopsy, the likelihood of findings consistent with CD was significantly higher in this group when compared to patients with negative serology or patients without serologic testing (Table 1, p < 0.001 for both comparisons, Fisher’s exact test). The overall incidence of histologic findings consistent with CD in the entire cohort was 3% (44/1432).

**Table 1:** Serologic testing in patients sent for duodenal biopsy

		**Biopsy result**			
	**N**	**Celiac**	**IVA**	**Other**	**NEG**
**No serology prior to biopsy**	977	1.6%	6.2%	7.6%	84.5%
**Pre-biopsy serology**					
Positive serology	68	35.3%	10.3%	2.9%	51.5%
Negative serology	387	1.0%	4.4%	8.3%	95.3%

N = total number of biopsies in each serologic category. Biopsy results refer only to findings from the duodenal biopsy. IVA = intact villous architecture with intra-epithelial lymphocytes. Findings consistent with celiac disease were significantly higher in patients with positive pre-biopsy serologic results when compared to either group (p < 0.001, Fisher’s exact test).

**Table 2:** Indication for endoscopy in patients where serologic testing was performed

**Indication for endoscopy**	**# Occurrences**	**% Patients**
Abdominal pain	260	52%
Diarrhea	99	20%
Reflux-type symptoms	91	18%
Nausea/vomiting	67	13%
Workup of celiac disease	53	11%
Weight loss	37	7%
Anemia	23	5%
GI bleeding	15	3%
Other	28	6%

Results are summarized for 504 patients where an indication was listed in the endoscopy report. For cases with multiple indications, each indication was listed as a separate occurrence. Only cases where celiac disease was specifically mentioned in the endoscopy report were counted as “workup for celiac disease”.

**Figure 2:**
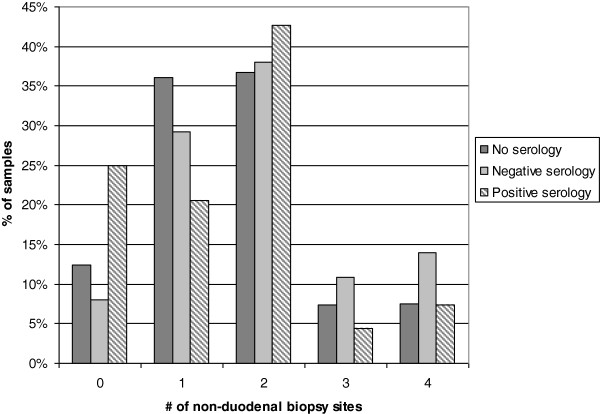
**Influence of serology results on number of biopsy sites.** Non-duodenal biopsy sites included esophagus, stomach, ileum, and colon. Patients were grouped according to pre-biopsy serology results as described in methods. The frequency of duodenal-only biopsy was significantly higher in patients with positive serology than in other groups (p < 0.01, chi-squared).

To understand why such a high proportion of these biopsies were sent in the absence of serologic findings, we looked for the presence of additional biopsy locations submitted concurrently with the duodenal biopsy. Virtually all duodenal biopsies in this study (88%) were submitted as part of a multi-site GI workup which included biopsies from at least one other location. The most common combinations were stomach and duodenum (26%) and stomach, esophagus, and duodenum (24%). The presence of a positive serology did seem to influence the decision to limit biopsy to the duodenum, as patients with a positive celiac serology result had a slightly lower number of biopsy sites (average 2.5 vs 2.9 for negative serology) and were the most likely group to have only a single sample submitted (Figure 2). However, even in patients with positive serology, multiple biopsy sites were a common occurrence.

Given that serologic status correlated well with the presence of celiac disease on biopsy, we examined the diagnostic yield of additional biopsy sites in these patients. We first evaluated the likelihood of significant findings in non-duodenal sites of patients with positive celiac serology. 58% of patients with positive celiac serology had findings noted in at least one non-duodenal site (Table 3). Although the majority of these findings were non-specific inflammatory changes, significant celiac-related disease findings such as lymphocytic gastritis and colitis were noted in non-duodenal sites for three patients. These data are in line with previous publications which note an increased rate of such findings in CD patients [22,23]. Conversely, in patients who had duodenal sections submitted despite having negative celiac serologic results only 14% had abnormal histology, with the most common findings being IVA-IEL and various presentations of non-specific duodenitis (Table 2). Villous atrophy was noted in four serology-negative cases, which included one case of collagenous sprue and two patients with known CD being evaluated for response to a gluten-free diet.

**Table 3:** Non-celiac findings in patients with multiple biopsy sites

**Non-duodenal findings in patients with positive pre-biopsy serology**				**Duodenal findings in patients with negative pre-biopsy serology**		
Finding	Site	N	%	Finding	N	%
Chronic gastritis	S	18	26%	IVA - IEL	17	4%
Active esophagitis	E	3	4%	Focal active inflammation	15	4%
Lymphocytic gastritis	S	2	3%	Peptic injury	11	3%
Helicobacter pylori	S	2	3%	Celiac disease	4	1%
Other gastric	S	2	3%	Chronic duodenitis	3	1%
Reflux, EE	E	2	3%	Intramucosal granulomas	2	1%
Ilietis	I	2	3%	Ulcer	1	0.3%
Active colitis	C	2	3%	Mild increased lymphocytes	1	0.3%
Other colon	C	2	3%			
Lymphocytic colitis	C	1	1%			

Results were tabulated for 409 patients with pre-biopsy serologic results and multiple biopsy sites. S = stomach. E = esophagus. I = ileum. C = colon. “Other gastric” includes erosive gastritis and a gastric polyp. “Other colon” includes tubular adenoma and mucosal prolapse. % is based on the number of patients in each serologic category.

**Figure 3:**
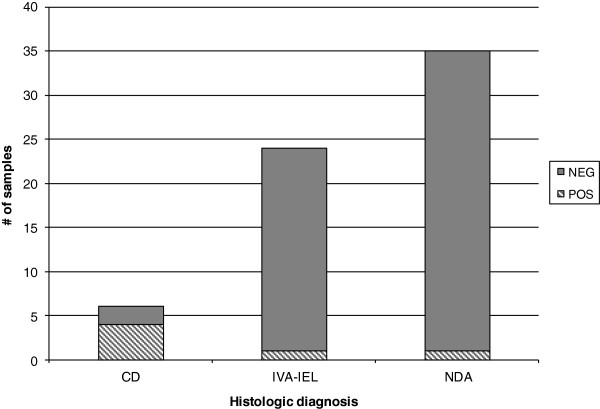
**Post-biopsy serologic results in patients biopsied for celiac disease.** Patients are grouped into findings consistent with celiac disease, partial villous atrophy with intraepithelial lymphocytes, or no duodenal disease detected. Positive serologic findings were rarely seen in patients without characteristic histology. The two exceptions were both patients who had an indeterminate serology result for a single marker (one tTG IgA, one dGDN IgA).

Finally, we identified a subset of 65 patients where serology had been ordered after the biopsy was performed. The majority of these were patients with indeterminate (IVA-IEL) or negative histologic findings. In these patients, positive post-biopsy serologic findings were almost entirely limited to patients who had characteristic celiac disease findings on the biopsy (Figure 3).

## Discussion

Evaluation for celiac disease is one of the most common reasons for duodenal biopsy. Positive findings in these biopsies are relatively uncommon, despite the widespread availability of screening assays for celiac-associated autoantibodies. The data presented here suggests that this is because the vast majority of duodenal samples being submitted for “rule out celiac” are not targeted biopsies driven by positive serologic results, but rather are part of a multi-site sampling strategy for a larger GI workup. In this type of clinical application, the diagnostic yield will necessarily be low. One limitation of the study is that because the analysis was based on EMR results, we cannot rule out the possibility that some patients were tested for celiac serology prior to referral to our system. However, these data suggest that when serologic results are available prior to biopsy, the information can be used to guide potential sampling strategies.

In patients with positive celiac serology results, non-duodenal findings were present in better than half of the patients. While many of these were non-specific changes such as chronic gastritis, significant findings such as lymphocytic gastritis and colitis were present in several patients. Because these entities are more common in patients with celiac disease [23], the endoscopist may wish to procure additional biopsies from sites such as stomach and colon in patients who have a positive serologic result prior to biopsy. In contrast, the value of duodenal biopsy in patients with a negative pre-biopsy serology is less clear. Histologic findings consistent with celiac disease were very uncommon in patients with negative serologies. One potential limitation of this study is that the frequency of duodenal bulb sampling was not noted, which could potentially lead to underdiagnosis of celiac disease in patients who were not adequately sampled. In the four patients where villous atrophy was observed despite a negative pre-biopsy serology, two were known CD patients, while a third patient had collagenous sprue, a variant of duodenal disease not associated with positive serology [24]. The majority of duodenal findings in this cohort were non-specific duodenitis or IVA-IEL. Based on chart review, no patients with IVA-IEL and negative serology were ultimately determined to have celiac disease. This suggests that in the setting of negative serologic studies, duodenal biopsies rarely provide clinically useful information that support the diagnosis of celiac disease.

## Conclusion

The decision to pursue a duodenal biopsy on a patient involves both clinical and serological factors, and the presence of high risk symptoms such as anemia or diarrhea is sufficient cause for biopsy in many published recommendations for the workup of celiac disease [25]. In addition, some authors have advocated that duodenal biopsy should routinely be performed in patients undergoing endoscopy for GERD [26]. However, in lower-risk patients the use of pre-endoscopy serology has been advocated as a tool that could optimize the decision to biopsy without reducing clinical sensitivity [27]. The data shown here support this contention, but suggest that this approach has not been widely adopted when deciding to pursue duodenal biopsy. In patients where serologic data is available, the results can help in the selection of GI locations to include in a multi-site biopsy strategy. The expanded use of serology to screen patients sent for endoscopy could potentially reduce the operational expenses associated with processing and evaluating large numbers of negative biopsies, resulting in more cost-effective treatment of these patients.

## Competing interest

The authors have no competing interest to disclose.

## Authors’ contributions

HW performed chart review and clinical classification of patient results, and drafted substantial portions of the primary manuscript. WH provided review of pathologic report findings and key suggestions on the analytical approach for the anatomic pathology portions of the manuscript. TD conceived of the study, participated in study design and primary data analysis of serologic results, and prepared the initial draft of the manuscript. All authors participated in revision and preparation of the final manuscript. All authors read and approved the final manuscript.

## Pre-publication history

The pre-publication history for this paper can be accessed here:

http://www.biomedcentral.com/1471-230X/13/156/prepub
